# Heritable CRISPR/Cas9-Mediated Genome Editing in the Yellow Fever Mosquito, *Aedes aegypti*

**DOI:** 10.1371/journal.pone.0122353

**Published:** 2015-03-27

**Authors:** Shengzhang Dong, Jingyi Lin, Nicole L. Held, Rollie J. Clem, A. Lorena Passarelli, Alexander W. E. Franz

**Affiliations:** 1 Department of Veterinary Pathobiology, University of Missouri, Columbia, Missouri, United States of America; 2 Division of Biology, Kansas State University, Manhattan, Kansas, United States of America; Louisiana State University Health Sciences Center, UNITED STATES

## Abstract

*In vivo* targeted gene disruption is a powerful tool to study gene function. Thus far, two tools for genome editing in *Aedes aegypti* have been applied, zinc-finger nucleases (ZFN) and transcription activator-like effector nucleases (TALEN). As a promising alternative to ZFN and TALEN, which are difficult to produce and validate using standard molecular biological techniques, the clustered regularly interspaced short palindromic repeats/CRISPR-associated sequence 9 (CRISPR/Cas9) system has recently been discovered as a "do-it-yourself" genome editing tool. Here, we describe the use of CRISPR/Cas9 in the mosquito vector, *Aedes aegypti*. In a transgenic mosquito line expressing both Dsred and enhanced cyan fluorescent protein (ECFP) from the eye tissue-specific 3xP3 promoter in separated but tightly linked expression cassettes, we targeted the ECFP nucleotide sequence for disruption. When supplying the Cas9 enzyme and two sgRNAs targeting different regions of the ECFP gene as *in vitro* transcribed mRNAs for germline transformation, we recovered four different G1 pools (5.5% knockout efficiency) where individuals still expressed DsRed but no longer ECFP. PCR amplification, cloning, and sequencing of PCR amplicons revealed indels in the ECFP target gene ranging from 2-27 nucleotides. These results show for the first time that CRISPR/Cas9 mediated gene editing is achievable in *Ae*. *aegypti*, paving the way for further functional genomics related studies in this mosquito species.

## Introduction

The yellow fever mosquito, *Aedes aegypti*, is the principal vector for important arboviruses such as yellow fever, dengue, and chikungunya viruses, which cause significant mortality and morbidity among humans living in tropical regions of the world [1, 2]. Major research efforts aim at understanding the genetics of vector competence for arboviruses in *Ae*. *aegypti* to explore novel ways to interrupt viral disease cycles [3]. Investigating the genetics of vector competence relies on the study of gene function. An important aspect when studying gene function is the ability to stably disrupt a gene-of-interest in a target-specific manner. Several targeted genome editing tools such as homologous recombination, zinc finger nucleases (ZFN) and transcription activator-like effector nucleases (TALEN) have been extensively used for the model insects *Drosophila melanogaster* and/or *Bombyx mori* [4–8]. Successful applications of ZFN and TALEN have been also described for targeted genome editing in mosquitoes [9–13]. Both systems involve specifically tailored DNA binding proteins to introduce double-strand breaks at the chosen target site of the host genome, leading to gene-knockout. ZFN and especially TALEN are highly effective; however, a major disadvantage is the fact that it is time-consuming and complicated to engineer and validate target gene-specific ZFN or TALEN tools in a standard laboratory. Consequently, most researchers purchase ZFN or TALEN reagents as custom-made tools from specialized, commercial sources.

A promising novel alternative is the clustered regularly interspaced short palindromic repeats/CRISPR-associated sequence 9 (CRISPR/Cas9) system, which has recently been discovered as a true "do-it-yourself" genome editing tool. Similar to ZFN and TALEN, the CRISPR/Cas9 system has been shown to be an efficient tool for genome editing in model organisms such as nematode, *Drosophila*, zebrafish, rat, mouse, and also in *B*. *mori* [14–21]. CRISPR/Cas9 was discovered as a prokaryotic immunity-like system in bacteria and archaea [22–27]. Type II CRISPR/Cas9 uses a CRISPR RNA (crRNA) and a transactivating RNA (tracrRNA) to guide the Cas9 DNA endonuclease to induce site-specific dsDNA cleavage [28, 29]. Target specificity of Cas9 is encoded by a 20-nucleotide (nt) spacer sequence in the crRNA, which pairs with the tracrRNA to direct the endonuclease to the complementary target site in the genome [28]. In *Drosophila*, a two-component system has been shown to be effective, in which crRNA and tracrRNA are fused into a single RNA called synthetic guide RNA (sgRNA). An essential requirement for efficient binding of the Cas9/sgRNA complex to the genomic target DNA is the presence in the target sequence of a short protospacer adjacent motif (PAM) adjacent to the 20 nt spacer sequence. The PAM typically consists of the 3 nt motif NGG (with N being A, C, G, or T) [29]. Thus, CRISPR/Cas9 based genome editing tools can be easily designed and generated, since a sgRNA with 20 nt target sequence identity adjacent to a 3 nt PAM is all that is needed for recognition of the target DNA sequence. Cas9/sgRNA-mediated double-strand breakage of the target DNA is recognized and repaired by the cellular DNA repair machinery via non-homologous end joining (NHEJ) typically resulting in short nucleotide deletions or insertions (indels), which disrupt the target gene.

As a proof-of-principle, we describe for the first time the use of CRISPR/Cas9 to stably disrupt a gene-of-interest in the mosquito vector, *Ae*. *aegypti*. We tested different CRISPR/Cas9 constructs with the aim to disrupt the coding sequence of the enhanced cyan fluorescent protein (ECFP) gene in a transgenic mosquito line, which expresses both ECFP and Dsred from the eye-specific 3xP3 promoter. Successful disruption of the marker gene demonstrated that the CRISPR/Cas9 system is a functional tool for targeted gene disruption in *Ae*. *aegypti*, although we found that the overall efficiency of the system appears to be lower in this insect species than what has been reported for *Drosophila* or *B*. *mori*.

## Materials and Methods

### Mosquitoes

**Fig 1:**
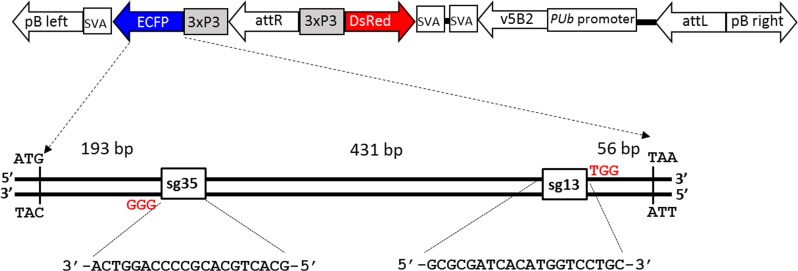
Schematic representation of the transgene in *Ae. aegypti* line PUbB2 P61 and the ECFP gene depicting sg35 and sg13 target sites. A single copy of the transgene is shown. The protospacer adjacent motifs (PAM) are indicated in red. Abbreviations: pB left, pB right = *piggyBac* transposon left or right arm; svA = SV40 polyA signal; ECFP = cyan fluorescent protein gene; 3xP3 = eye-specific synthetic promoter; attR, attL = right or left PhiC31 attachment site; DsRed = red fluorescent protein gene; v5B2 = Flockhouse virus B2 gene; *PUb* = *Ae*. *aegypti* poly-ubiquitin promoter.

*Ae*. *aegypti* recipients for CRISPR/Cas9-mediated gene disruption were hybrids resulting from a cross between the Higgs white eye strain (HWE) [30] and transgenic line PubB2 P61 [31, 32]. PubB2 P61 mosquitoes harbor two *piggyBac* transposable element (TE) integrations. Each copy of the *piggyBac* transgene contains two separate fluorescent eye marker expression cassettes, DsRed and ECFP, each under control of the 3xP3 promoter (Fig. 1). Both eye marker expression cassettes are physically closely linked based on *PhiC31*-mediated recombination, in which ECFP originates from docking strain attP26 and DsRed from the *attB*-site containing donor plasmid. To maintain the transgenic line in a double-hemizygous state, inbred PUbB2 P61 mosquitoes were outcrossed to the non-transgenic HWE recipient strain.

### Plasmid constructs

Plasmid phsp70-Cas9 containing the coding sequence (CDS) of Cas9 was obtained from Addgene (https://www.addgene.org/45945) [33]. Two different Cas9 expression vectors were derived from this plasmid: PUb/Cas9/SV40A and hsp70/Cas9/SV40A. To create PUb/Cas9/SV40A, the Cas9 CDS of phsp70-Cas9 was inserted into pSLfa1180fa-PUb/SV40A [32] using restriction enzymes *Not*I and *Nhe*I. Therefore the Cas9 CDS was PCR-amplified using a forward primer containing the *Not*I site and a reverse primer containing the *Nhe*I site. PCR amplification was conducted using AccuPrime proof-read polymerase (Invitrogen, Life Technologies, Carlsbad CA). Plasmid hsp70/Cas9/SV40A was generated by exchanging the hsp70 3’UTR for the SV40A polyadenylation sequence using restriction enzymes *Xba*I and *BamH*I. As described above, matching restriction sites were added to the cDNA insert via proof-read polymerase PCR.

DrU6.sgRNA constructs were based on pU6-*Bbs*I-chiRNA, which was obtained from Addgene (https://www.addgene.org/45946). ECFP-targeting, guide sequence containing cDNAs were inserted into plasmid DrU6.sgRNA via *Bbs*I restriction sites. For each guide sequence, a separate *Ae*. *aegypti* U6 promoter (AeU6).sgRNA construct was generated as a custom-made cDNA molecule (IDT-DNA, Coralville, IA), which was then inserted into pSLfa11280fa using *Sac*I and *Sma*I. The nucleotide sequence of the *Ae*. *aegypti* U6 promoter (AeU6) is: 5’-GAATGAAATCGCCCATCGAGTTGATACGTCCATCCATCGCTAGAACCGCGTTCGCTGTAGAAGACTATATAAGAGCAGAGGCAAGAGTAGTGAAAT-3’ [34].

ECFP-targeting guide sequences were based on the ECFP CDS (GenBank accession: KJ081792.1) and identified using the ZiFiT Targeter Version 4.2 design tool (http://zifit.partners.org/ZiFiT). Suggested guide sequences were validated for unique target specificity by blasting against the *Ae*. *aegypti* genome (AaegL.3.2.) (https://www.vectorbase.org/organisms/aedes-aegypti). Two guide RNA sequences were chosen: sg13 5’-GCGCGATCACATGGTCCTGC-3’ and sg35 5’-GCACTGCACGCCCCAGGTCA-3’. sg13 targeted ECFP between nucleotide positions 645–664 of the sense strand (with TGG as PAM motif) and sg35 targeted ECFP between nucleotide positions 213–194 of the antisense strand (with GGG as PAM motif) (Fig. 1; S1A Fig).

### Expressing Cas9 and chimeric RNAs from *in vitro* transcribed RNAs

For Cas9 mRNA *in vitro* transcription, plasmid MLM3613 was obtained from Addgene (https://www.addgene.org/42251) and was linearized using *Pme*I. Linearized plasmid was *in vitro* transcribed using mMESSAGE mMACHINE T7 ULTRA kit (Ambion, Life Technologies) following the protocol of the manufacturer. After transcription, a poly(A) tail was added to the 3’ end of the capped mRNA using the Poly(A) Tailing Kit (Ambion, Life Technologies). The Cas9 mRNA was purified with MEGAclear Transcription Clean-Up Kit (Ambion, Life Technologies). As template for guide RNA expression from the T7 RNA promoter, plasmid DR274 was obtained from Addgene (https://www.addgene.org/42250). ECFP targeting guide sequences 13sgRNA and 35sgRNA were inserted into DR274 as described above. sgRNAs were *in vitro* transcribed from the *Dra*I-digested sgRNA expression vectors using the MAXIscript T7 *in vitro* Transcription Kit (Ambion, Life Technologies). Resulting sgRNAs were purified with MEGAclear Transcription Clean-Up Kit (Ambion, Life Technologies).

### Microinjections, outcrossing, pooling of mosquitoes

**Fig 2:**
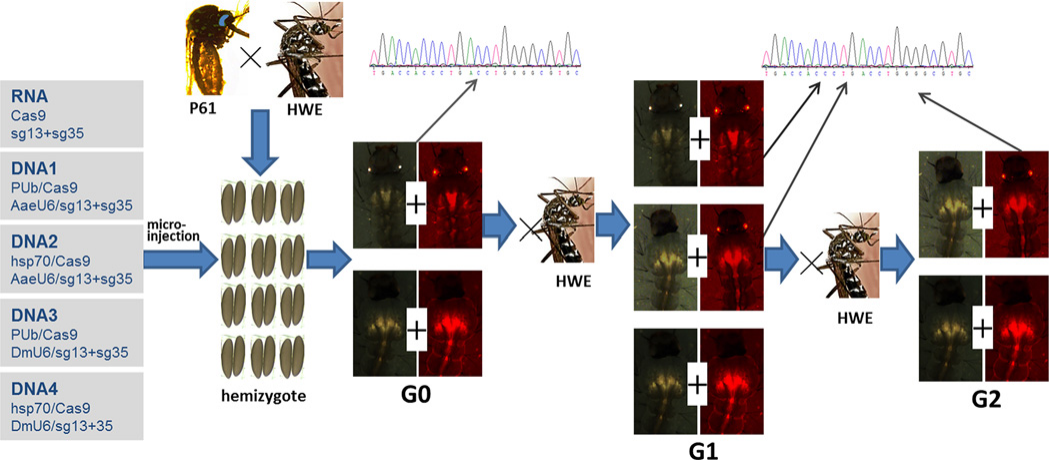
Flowchart showing generation and identification of ECFP knockout mutants based on transgenic PUbB2 P61 x HWE hybrids. Five groups of Cas9/sgRNA constructs were each micro-injected into ~500–700 double-hemizygous embryos (G_0_). All surviving G_0_ individuals were outcrossed to HWE and pooled. All G_1_ individuals of each pool were screened for the ECFP knockout phenotype. Pools containing individuals with ECFP knockout phenotype were again outcrossed to HWE to generate G_2_. DNA was extracted and sequenced from G_0_ adults and from G_1_ and G_2_ larvae showing ECFP knockout phenotype and also from groups of G_1_ larvae, which did not show a mutant phenotype.

Purified Cas9 mRNA and each sgRNA, 13sgRNA and 35sgRNA, were mixed to final concentrations of 1 μg/μl and 50 ng/μl respectively, prior to injection into PUbB2 P61 x HWE hybrid embryos. Cas9-expressing plasmid DNA and sgRNA encoding plasmids were injected at final concentrations of 500 ng/μl and 150 ng/μl, respectively. The following combinations were set up: RNA: T7/Cas9+T7 sg13+T7 sg35; DNA1: PUb/Cas9+Ae(des)U6 sg13+AeU6 sg35; DNA2: hsp70/Cas9+AeU6 sg13+AeU6 sg35; DNA3: PUb/Cas9+Dm(*Drosophila*)U6 sg13+DmU6 sg35; DNA4: hsp70/Cas9+DmU6 sg13+DmU6 sg35 (Fig. 2).

Plasmid DNAs were diluted in 2X microinjection buffer [5 mM KCl, 0.1 mM NaH_2_PO_4_ (pH 6.8)] [35]. Embryo microinjections were performed as previously described [32, 35, 36]. Five days post-injection, eggs were hatched and survival rates recorded (Table 1). Adult G_0_ were visually screened for loss of eye-specific ECFP expression under a Leica M10 stereomicroscope equipped with a fluorescent light source and specific filter sets. Surviving G_0_ males were singly outcrossed to 15 HWE females and surviving G_0_ females were pooled in groups of 4 to 14 and outcrossed to three HWE males each. Following a 2-day mating period, crosses based on G_0_ males were pooled in groups of two to three in order to reduce the number of required bloodfeeds. A few of the RNA-injected G_0_ individuals were not pooled following outcrossing to HWE males; instead they were maintained as families (a single injected G_0_ founder x HWE). Mosquitoes were artificially bloodfed with defibrinated sheep blood (Colorado Serum Co, Denver, CO) as described [31, 36]. At least two eggliners were produced from each pool/family.

**Table 1:** Transformation data of PUbB2 P61 x HWE embryos injected with different CRISPR/Cas9 constructs

	RNA	DNA1	DNA2	DNA3	DNA4
No. of embryos injected	626	685	602	529	605
No. of G_0_ survivors	27♀, 46♂	36♀, 38♂	14♀, 13♂	39♀, 35♂	34♀, 36♂
No. of pools	7♀, 16♂	5♀, 16♂	1♀, 5♂	4♀, 11♂	3♀, 12♂
No. of G_1_ KO pools	2♀, 2♂	0	0	0	0
Estimated KO efficiency	5.5%				

KO: knockout.

### DNA Sequencing of genomic regions targeted by CRISPR/Cas9

Each target locus of the ECFP gene was PCR-amplified from genomic DNA of individual adults (G_0_) or pools of 5 to 20 G_1_ or G_2_ larvae. PCR products were either sequenced directly or cloned into a plasmid vector using the pCR4-TOPO TA cloning kit (Invitrogen, Life Technologies) prior to sequencing. From each *Escherichia coli* transformation, at least 10 colonies were picked and prepared for sequencing at the University of Missouri DNA Core. Mutated alleles were identified by sequence comparison with the original ECFP nucleotide sequence.

## Results

### Embryo microinjection and survival rates

Numbers of microinjected eggs for each CRISPR/Cas9 construct ranged from 529 (DNA3) to 685 (DNA1) (Table 1). Survival rates were lowest for DNA2 (4.5%) and highest for RNA (11.7%). With the exception of the RNA-injected eggs where only 1/3 of the survivors were female, sex ratios were balanced in the other treatments. Only three out of 23 RNA injected mosquito pools and one family (based on a single female G_0_ founder) produced G_1_ individuals showing ECFP knockout. Assuming that in each of the four pools only a single G_0_ individual was mutant (see below), we estimated a target gene knockout efficiency of 5.5%.

### Visual screening for ECFP knockout phenotypes

**Fig 3:**
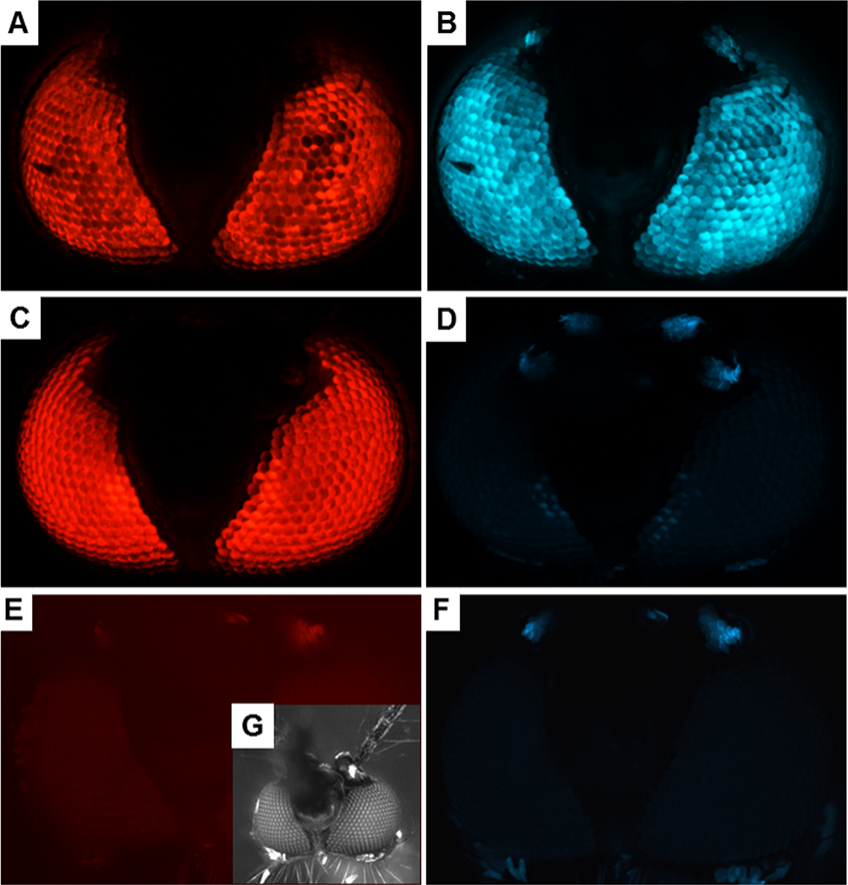
Eye marker expression in PUbB2 P61 x HWE mosquitoes before and after CRISPR/Cas9 mediated ECFP knockout. Eyes were viewed under a fluorescent stereo microscope (Leica M205) equipped with DsRed (A, C, E) or ECFP (B, D, F) specific filter sets. (A, B) Eyes of a PUbB2 P61 x HWE female. (C, D) Eyes of a (PUbB2 P61 x HWE) P41 female (G_1_) originating from an embryo which had been injected with *in vitro* transcribed RNAs encoding Cas9 and two ECFP targeting sgRNAs, sg13 and sg35. (E, F) Eyes of a HWE female. (G) Eyes of the HWE female under bright field.

None of the RNA- or DNA-injected G_0_ survivors showed loss of eye-specific ECFP expression. Three pools of the RNA-injected mosquitoes, P41, P49, P55, and one family, F82, generated G_1_ larvae amongst which eye-specific ECFP expression was no longer visible, even though these larvae strongly expressed DsRed. These phenotypes were maintained in eyes of adults (Fig. 3). However, we did not detect any G_1_ larvae with ECFP knockout phenotype among those pools injected with DNA1, DNA2, DNA3, or DNA4.

The presence of an ECFP knockout phenotype in double-hemizygous G_1_ and its absence among double- hemizygous G_0_ individuals supports the conclusion that in G_0_ only one ECFP allele was disrupted by CRISPR/Cas9. This conclusion is further supported by the segregation patterns among G_1_ of pools P41, P49, P55, and family F82, which all contained individuals with ECFP phenotypes (Table 2).

**Table 2:** Proportion of different eye marker phenotypes among G_1_ and G_2_ larvae of pools P41, P49, P55, and family F82.

Phenotype	Pool 41 3♂×40 HWE♀	Pool 49 3♂×40 HWE♀	Pool 55 9♀×21 HWE♂	Family 82 1♀×3 HWE♂
G_1_ progeny resulting from **1. bloodfeed** of G_0_ hybrids*				
Dsred positive, ECFP positive	190	13	131	28
DsRed positive, ECFP negative	**70 (13%)**	**158 (46%)**	**0**	**3 (5%)**
DsRed negative, ECFP negative	294 (53%)	169 (50%)	161 (55%)	35 (53%)
Total	554	340	292	66
G_1_ progeny resulting from **2. bloodfeed** of G_0_ hybrids*				
Dsred positive, ECFP positive	170	11	247	33
DsRed positive, ECFP negative	**42 (10%)**	**89 (52%)**	**37 (8%)**	**9 (17%)**
DsRed negative, ECFP negative	190 (47%)	72 (42%)	175 (38%)	20 (38%)
Total	402	172	459	52
Outcrossed G_2_ progeny**				
Dsred positive, ECFP positive	0	0	0	0
DsRed positive, ECFP negative	**284 (50%)**	**325 (47%)**	**290 (49%)**	**54 (50%)**
DsRed negative, ECFP negative	286 (50%)	363 (53%)	296 (51%)	53 (50%)
Total	570	688	586	107

*PUBB2 P61 x HWE embryos (G_0_) were injected with CRISPR/Cas9 RNA constructs.

**G_2_ mutants x HWE.

Around 53% of the G_1_ PUbB2 P61 x HWE hybrids arising from the first parental bloodfeeding were non-transgenic, which follows the expected segregation pattern considering the transgene-associated fluorescent eye markers being dominant traits. Interestingly, the average proportion of non-transgenic progeny arising from the second parental bloodfeeding was only 42%. Proportions of G_1_ larvae showing knockout of ECFP varied between 5 and 46% (from first parental bloodmeal) and 8 and 52% (from second parental bloodmeal). Extreme observations were F82 with only a single female G_0_ founder and P49 based on three male G_0_ founders, in which <10% and >90%, respectively, of the transgenic G_1_ individuals had an ECFP knockout phenotype. Similar to the other pools, all G_1_ siblings of P49 showed a uniform indel variant. Thus, it is unlikely that more than one male founder of P49 carried the ECFP knockout genotype.

Outcrossing of the G_1_ mutants to HWE showed that the ECFP knockout mutations were stably inherited in the following generation. The proportion of individuals exhibiting the mutant phenotype amounted to around 50% in outcrossed G_2_ of P41, P49, P55, and F82 (Table 2).

### Genotypic analysis of ECFP knockout mosquitoes

**Fig 4:**
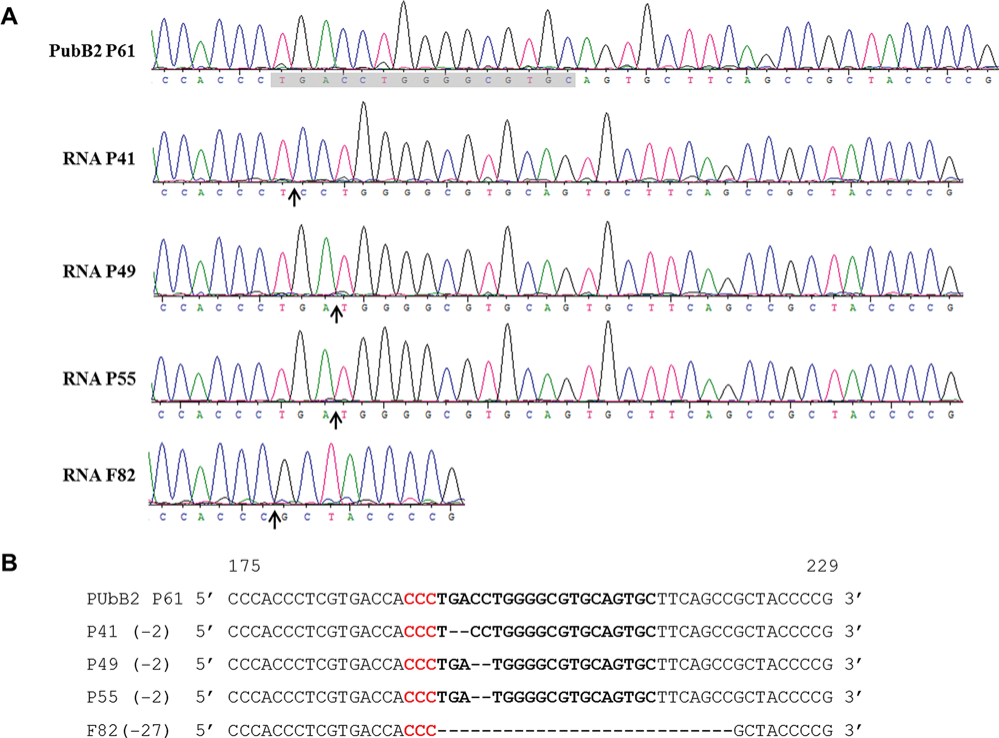
CRISPR/Cas9 mediated indels in the ECFP gene of PUBB2 P61 x HWE hybrids. (A) Sanger-sequencing trace data showing the regions of the indels in mutants P41, P49, P55, and F82. Arrows indicate the cleavage site; the sequence of guide RNA sg35 is shadowed. (B) Nucleotide sequence alignments showing indels. The ECFP nucleotide sequence is shown at the top with the sg35 target site in bold-type and the PAM sequence in red. Deletions are shown as dashes. cDNA fragments spanning each target site were PCR-amplified using genomic DNA from pooled larvae as template and cloned into the pCR4-TOPO TA vector. At least 10 cDNA clones per PCR amplicon were sequenced.

In pools P41, P49, and P55, CRISPR/Cas9 mediated targeting of the genome by sg35 resulted in a deletion of two nucleotides within the CDS of ECFP leading to a frameshift mutation within the gene (Fig. 4). In P49 and P55, both having an identical indel variant, two nucleotides were deleted 3 nt downstream of the PAM sequence, which is considered the typical cleavage site of Cas9 [28]. In P41, the deletion occurred 1 nt downstream of the PAM sequence. In F82, a 27 nt deletion adjacent to the PAM sequence resulting from sg35-mediated CRISPR/Cas9 genome targeting translated into the loss of the peptide LTWGVQCFS from the predicted ECFP protein. In the highly similar enhanced green fluorescent protein, the tripeptide T66Y67G68 (L**T** **Y** **G**VQCFS) is essential for forming the p-hydro-xybenzylidene-imidazolinone (HBI) chromophore within the central helix that runs through the center of the capped beta-barrel structure of the protein [37]. In general, cleavage patterns and size ranges of CRISPR/Cas9-mediated indels in the genomic DNA of *Ae*. *aegypti* were not fundamentally different from those observed in the genomes of *Drosophila* or *B*. *mori* [38–42]. In the outcrossed G_2_, the same indels were detected for P41, P49, P55, and F82 indicating that those knockout mutations were stable and heritable (data not shown).

Interestingly, only sg35 but not sg13 enabled ECFP-specific dsDNA cleavage even though both sgRNAs were selected using the same ZiFiT algorithm. Previously, it had been revealed that the efficiency of a sgRNA to bind to its target correlated with the GC content in its sequence 6 nt adjacent to its PAM [16]. However, both sgRNAs, sg13 and sg35, had the same level of GC content (67%) in their respective 6 nt sequences near the PAM. For sg35 no sequence similarity was detected in the genome of *Ae*. *aegypti* whereas for sg13, 14 of 20 nucleotides of the 5’ region of the sgRNA were identical to the CDS of gene AAEL007920 (though an adjacent PAM motif was absent in the gene sequence) (S1B Fig).

Sequencing of DNA fragments spanning the sgRNA target sites from 15 (out of 23) G_0_ individuals originating from the RNA injected embryos (Table 1) did not result in the detection of indels. Also, no indels were revealed when sequencing DNAs from G_1_ larvae (~20 larvae/sample) of 13 different pools including P41, P49, P55, and F82, which originated from RNA injected G_0_ embryos and expressed both eye markers (Fig. 2).

## Discussion

We targeted the eye marker gene ECFP in a double-hemizygous transgenic *Ae*. *aegypti* hybrid, PUbB2 P61 x HWE, expressing both DsRed and ECFP from the eye-specific 3xP3 promoter. Successful knockout of ECFP was predicted to result in individuals that express only the DsRed marker. Two sgRNA sequences were designed with the intention to cleave the ECFP gene simultaneously at two sites, separated by 470 bp, to obtain a truncated ECFP gene in the mutated progeny. According to reports from the literature, we tested several construct designs to identify a procedure that yielded maximal efficiency in *Ae*. *aegypti*. Using the two-plasmid component system for germline-specific expression of Cas9 (non-codon optimized for *Ae*. *aegypti*) and sgRNAs, construct designs included the *AePUb* or *Drosophila hsp70* promoters driving expression of Cas9 and AeU6 or *Drosophila* U6 promoters for sgRNA expression. Alternatively, we transformed embryos with separate *in vitro* transcribed mRNAs encoding Cas9 and the two sgRNAs [15, 38]. Interestingly, only the *in vitro* transcribed mRNAs resulted in the generation of ECFP knockout mutants. We think that the failure of the plasmid DNA constructs to generate ECFP knockout mutants could be due to two major reasons. First, the plasmid DNA systems may have had too low efficiencies in *Ae*. *aegypti* compared to *B*. *mori* or *Drosophila* so that a number of 500–700 injected embryos was generally too low to recover mutants. Alternatively, the promoters of the constructs may not have driven high enough expression of the CRISPR/Cas9 system in *Ae*. *aegypti*. However, in conjunction with applications other than CRISPR/Cas9, both *Drosophila hsp70* and *AePUb* promoters have been shown to efficiently drive gene expression in *Ae*. *aegypti* embryos [9, 35, 36], making this an unlikely possibility. We only tested a single AeU6 promoter in our assay, which had been characterized and successfully used in an earlier study [34]. It is possible that this promoter might not be the optimal choice for the CRISPR/Cas9 system. Likewise, the *Drosophila* U6 promoter might not be functional in the mosquito germline.

In *Drosophila*, the *yellow* gene was knocked out with a germline mutation rate of up to 6% when using a two-plasmid component system in which Cas9 was expressed from the *Drosophila hsp70* promoter and a single sgRNA from *Drosophila* U6 [33, 39]. The germline mutation rate based on *yellow* mosaic phenotypes observed among the injected G_0_ increased to 66% (n = 52) when 2 sgRNAs were used to delete the coding sequence of *yellow* instead of a single sgRNA disrupting the gene at a single locus. Germline mutation rates in *B*. *mori* ranged between 17 and 30% when using plasmid DNAs to express Cas9 and sgRNAs from *B*. *mori*-specific promoters. The variation in mutation efficiency was strongly affected by the sequence of each of the three different sgRNAs, which were used to disrupt the *BmKu70* gene [40]. Another report using the CRISPR/Cas9 system in *B*. *mori* showed a mutation frequency of 15% in the *kynu-2* locus of *B*. *mori* embryos injected with plasmids expressing Cas9 and a single sgRNA [41].

When using *in vitro* transcribed mRNAs of Cas9 and sgRNAs, we obtained a germline mutation rate of 5.5%. This is in stark contrast to observations with *Drosophila* where knockout mutation rates reached 86% in G_0_ when expressing Cas9 and sgRNAs from *in vitro* transcribed mRNAs [15]. Bassett and colleagues [15] showed that reducing Cas9 mRNA concentrations in their embryo injection experiments while keeping sgRNA concentrations (50 ng/μl) constant, reduced the proportion of G_0_ *Drosophila* with mosaic phenotype from 86% (1 μg/μl Cas9 RNA) to 10% (0.13 μg/μl Cas9 RNA). In our experimental set-up with *Ae*. *aegypti*, we applied Cas9 mRNA and sgRNAs at concentrations of 1 μg/μl and 50 ng/μl, respectively, which in *Drosophila* led to a maximal mutation rate.

Contrary to the findings described above, we were not able to discover ECFP mosaic phenotypes among the G_0_ of *Ae*. *aegypti*. However, outcrossing to the non-transgenic HWE recipient strain resulted in G_1_ that produced the ECFP knockout phenotype (Table 2, Fig. 3). As stated above, we think that only one of two ECFP gene alleles was disrupted among G_0_ individuals of pools P41, P49, P55, and family F82. Thus, the dominant intact ECFP allele would produce an intact fluorescent blue-eye phenotype, which then would overshadow any mosaic expression pattern in the same tissue. We maintained the CRISPR/Cas9 recipient mosquito strain, PUbB2 P61 x HWE, in a hemizygous state because we were aware that this transgenic line harbors two transgene copies and we could not predict how efficiently the genome editing tool would target both alleles simultaneously instead of one. However, due to segregation of the G_1_, we could easily identify ECFP knockout mutants, which were stably maintained in outcrossed G_2_.

We designed two sgRNAs, 470 bp apart from each other, to delete a major portion of the ECFP gene. Surprisingly, sgRNA13 did not seem to bind to its target site to promote dsDNA breakage followed by NHEJ. Both sgRNAs were designed to recognize the same target gene, had similar GC contents and secondary structures and were selected by the same ZiFiT algorithm. Thus, it is unclear why sg13 was not functional. Other researchers have also noticed that various sgRNAs can differ substantially in their dsDNA cleavage efficiency [15, 16, 33]. Wei and colleagues [42] speculated that potential secondary structures or sequence motifs in sgRNA sequences may account for different target site recognition efficiencies, but these are still not well understood. Our observation could be an indication that sgRNAs may function less efficiently in the complex genomic environment of *Ae*. *aegypti* than in the condensed genome structure of *Drosophila*. Our results also indicate that more than one or two sgRNA sequences for each DNA target in *Ae*. *aegypti* may need to be tested. Thus, a multiplex gene targeting approach as successfully applied in *Drosophila* and *B*. *mori* to target several genes simultaneously or to delete larger fragments of genomic DNA may be more difficult to design for *Ae*. *aegypti* [17, 33, 41].

## Conclusions and Future Directions

We tested the efficacy of the CRISPR/Cas9 system in a transgenic mosquito line expressing two different eye markers, which allowed us to take advantage of a simple visual screening system for knockout mutants (presence of DsRed expression in absence of ECFP expression). Use of a rapid visual screening system was of great importance, because it was not foreseeable whether our CRISPR/Cas9 constructs would be functional in *Ae*. *aegypti* and if so, how efficient they would be. Since our transgenic line had two transgene copies, it complicated the screening process because mosaic-type mutants could not be identified among the G_0_ as may have been more likely in presence of a single transgene copy. Regardless, line PUbB2 P61 was the most suitable mosquito line we had available for this proof-of-principle study. Our study clearly demonstrates that CRISPR/Cas9-mediated gene editing is achievable in *Ae*. *aegypti*. Several observations suggest that the anticipated ease-of-use of the CRISPR/Cas9 system may be less efficient in this insect species: i) only one of two sgRNAs designed to target the same marker gene was functional indicating that it might be more difficult to predict successful sgRNA designs for use in *Ae*. *aegypti*; ii) only the *in vitro* transcribed mRNA Cas9 and sgRNA constructs, and not those based on expression plasmids enabled genome targeting and dsDNA cleavage in *Ae*. *aegypti*; iii) when using *in vitro* transcribed Cas9 mRNA and sgRNAs, mutation rates in *Ae*. *aegypti* were substantially lower than those observed in *Drosophila* and *B*. *mori*. However, it needs to be emphasized that these observations need further validation in subsequent experiments.

Future refinements aimed at enhancing Cas9/sgRNA expression levels and efficiencies could include the generation of transgenic driver strains of *Ae*. *aegypti* that would express the Cas9 gene in the germline from the *Ae-nanos* promoter, similar to proven strategies for *Drosophila* [16, 43–45]. Further, bicistronic expression of Cas9 and sgRNA from the same plasmid vector [46], or development of a tissue-specific CRISPR/Cas9-mediated conditional mutagenesis system by combining CRISPR/Cas9 with the UAS-Gal4 system [47] could potentially improve the efficacy of the CRISPR/Cas9 system in *Ae*. *aegypti*.

An important and novel application of the CRISPR/Cas9 system in *Ae*. *aegypti* would be the targeted disruption of an endogenous gene-of-interest in combination with knock-in of a selectable marker gene via homologous recombination. In view of such an experiment, we are now confident that *in vitro* transcribed mRNA-based CRISPR/Cas9 constructs are functional in *Ae*. *aegypti*.

## Supporting Information

S1 Fig (A) Sequence alignments of guide RNAs sg35 (blue/bold) and sg13 (burgundy/bold) with the nucleotide sequences of the fluorescent reporter genes ECFP, EGFP (enhanced green fluorescent protein), and DsRed. Nucleotide mismatches are shown in red. (B) Alignment of guide RNA sg13 with endogenous gene AAEL007920 of *Ae*. *aegypti*. Location where a hypothetical PAM sequence should be present is highlighted in blue. Nucleotide mismatches are shown in red.
(TIF)
