# Supplementary figures and images for: Heritable CRISPR/Cas9-Mediated Genome Editing in the Yellow Fever Mosquito, Aedes aegypti
Source: PLoS One. 2015 Mar 27;10(3):e0122353. doi: 10.1371/journal.pone.0122353 (PMC4376861; doi:10.1371/journal.pone.0122353)

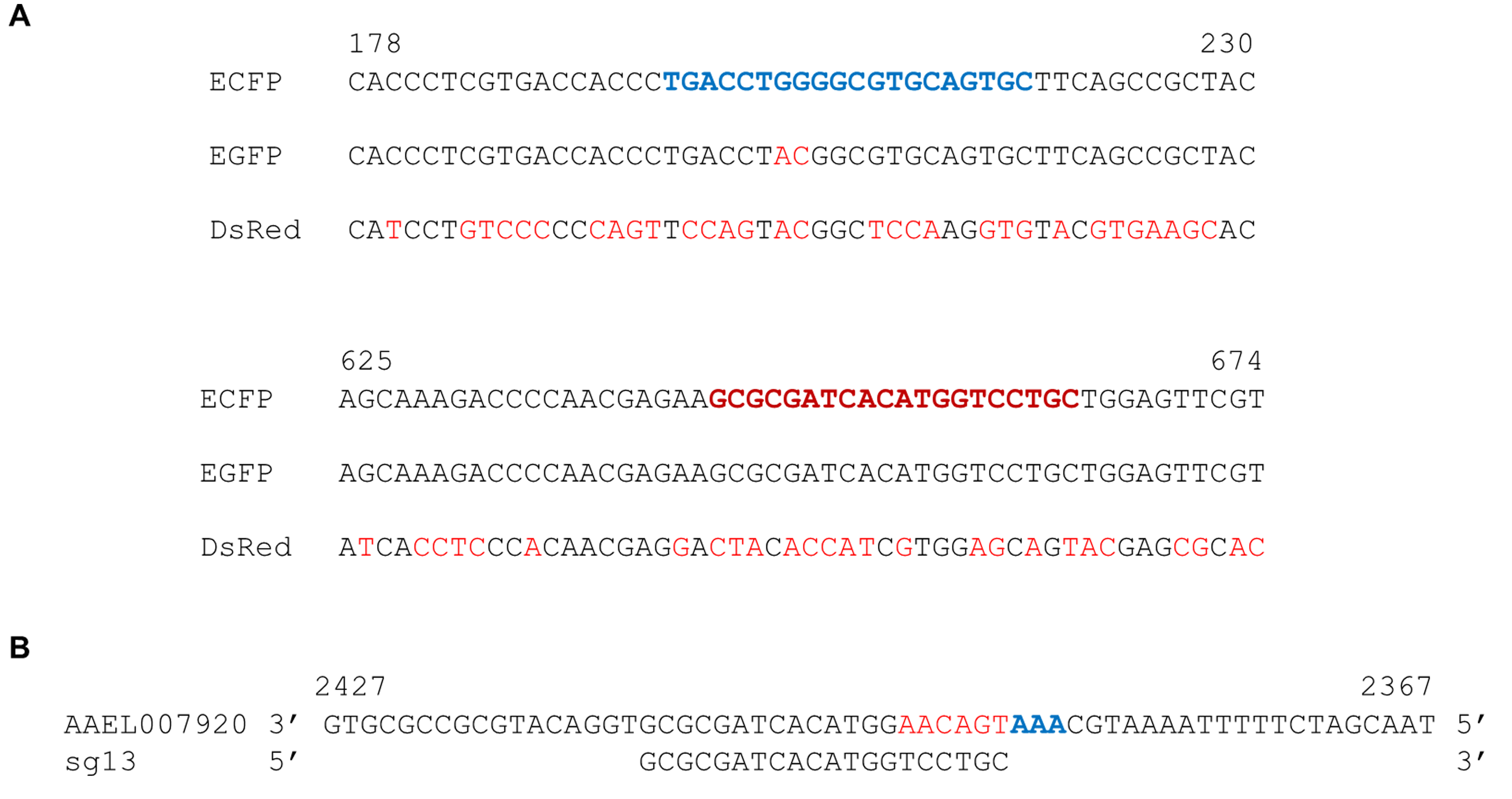

Supplement: S1 Fig — (A) Sequence alignments of guide RNAs sg35 (blue/bold) and sg13 (burgundy/bold) with the nucleotide sequences of the fluorescent reporter genes ECFP, EGFP (enhanced green fluorescent protein), and DsRed. Nucleotide mismatches are shown in red. (B) Alignment of guide RNA sg13 with endogenous gene AAEL007920 of Ae. aegypti. Location where a hypothetical PAM sequence should be present is highlighted in blue. Nucleotide mismatches are shown in red. (TIF) [file pone.0122353.s001.tif]
